# Steered molecular dynamic simulations of conformational lock of Cu, Zn-superoxide dismutase

**DOI:** 10.1038/s41598-019-40892-0

**Published:** 2019-03-13

**Authors:** Bao-Lin Xiao, Yan-Na Ning, Nan-Nan Niu, Di Li, Ali Akbar Moosavi-Movahedi, Nader Sheibani, Jun Hong

**Affiliations:** 1 School of Life Sciences, Henan University, JinMing Road, Kaifeng, 475000 China; 2 Institute of Biochemistry and Biophysics, University of Tehran, Enquelab Avenue, P.O. Box 13145-1384, Tehran, Iran; 3 Department of Ophthalmology and Visual Sciences and Biomedical Engineering, University of Wisconsin, School of Medicine and Public Health, Madison, WI 53726 USA; 4 Henan Engineering Laboratory for Mammary Bioreactor, School of Life Sciences, Henan University JinMing Road, Kaifeng, 475000 China

## Abstract

The conformational lock was a bio-thermodynamic theory to explain the characteristics of interfaces in oligomeric enzymes and their effects on catalytic activity. The previous studies on superoxide dismutases (Cu, Zn-SODs) showed that the dimeric structure contributed to the high catalytic efficiency and the stability. In this study, steered molecular dynamics simulations were used firstly to study the main interactions between two subunits of Cu, Zn-SODs. The decomposition process study showed that there were not only four pairs of hydrogen bonds but also twenty-five residue pairs participating hydrophobic interactions between A and B chains of SOD, and van der Waals interactions occupied a dominant position among these residue pairs. Moreover, the residue pairs of hydrogen bonds played a major role in maintaining the protein conformation. The analysis of the energy and conformational changes in the SMD simulation showed that there were two groups (two conformational locks) between A and B chains of SOD. The first group consisted of one hydrogen-bond residues pair and seven hydrophobic interactions residues pairs with a total average energy of −30.10 KJ/mol, and the second group of three hydrogen-bond residues pair and eighteen hydrophobic interactions residues pairs formed with a total average energy of −115.23 KJ/mol.

## Introduction

The conformational lock, which was firstly proposed by Poltorak in 1998, was a bio-thermodynamic theory to explain the characteristics of interfaces in oligomeric enzymes and their effects on catalytic activity. This theory could be applied by using the structural and kinetics data, respectively. According to this Poltorak’s theory, a simple thermal dissociation could be expressed as Eq. 1:

1 $${E}_{2}\iff 2{E}_{1}\Rightarrow 2{E}_{d}$$

E_2_ was an active dimer form of enzyme, E_1_ and E_d_ were the monomers for the reversible transformation of initial structure and for denatured form of irreversible transformation of E_1_, respectively. The E_d_ could not reunite and form an active enzyme (E_2_). Generally, there were several intermediate active forms of dimer enzyme (Eq. 2):

2 $${E}_{2}\iff {{E}_{2}}^{1}\iff {{E}_{2}}^{2}\iff {{E}_{2}}^{3}\mathrm{...}{{E}_{2}}^{m}\iff 2{E}_{1}\Rightarrow 2{E}_{d}$$

It was supposed that the real number (m) of intermediates of catalytically active protein was always greater than a certain quantity (n). Here, n represented different active forms of the dimer enzyme, and could be obtained from an empirical Eqs 3 and 4:

3 $$n=\frac{0.13+\delta }{0.13-0.05\delta }$$

4 $$\delta =R-1$$

where, δ was obtained from the kinetic plot of residue active versus time and depending only on the number of steps (n) before loss of activity of enzyme. These made it possible to estimate the minimal number of steps for a dimer enzyme in the process of thermal dissociation into inactive monomers. These phenomenons of interprotein contacts with possible partial breaks were also supposed as “conformational lock”. From then on, various dimer enzymes from different sources have been investigated that in both methods were in reasonable agreement. Conformational lock theory might provide a special explanation about the process of thermal dissociation and denaturation by obtaining the certain quantity of real number of intermediate active forms (number of conformational locks) of an oligomeric protein^1–6^. However, there were many disadvantages in those research methods on conformational lock, such as tedious time and large amounts of protein consumption, high cost, repeated operation, and large experimental error. Particularly, the decomposition process could not be dynamically represented.

Superoxide dismutases (SODs) exist in the periplasm of several bacterial species and the cytoplasm of all the eukaryotic cells^7,8^. SODs catalyze the dismutation of the superoxide anion at a diffusion-limited rate enhanced by electrostatic guidance of the substrate to the active site^9^. Eukaryotic Cu, Zn-SODs are homodimers that contain one atom of zinc and one atom of copper per subunit. Since the discovery of Cu, Zn-SOD, there has been serious in understanding how the dimeric structure contributes to the high catalytic efficiency and the remarkable stability of this class of enzymes. The monomeric Cu, Zn-SODs have very low catalytic activity and great changes in optical properties^10,11^. These dramatic changes probably reflect changes in the tertiary structure consequent to rearrangements of the solvent-exposed hydrophobic dimer interface.

In our previous study^5^, we had put forward a mechanism of Cu, Zn-SOD denaturation based on structural data, thermal dissociation and conformational lock. Biochemical calculation was applied to achieve a putative mechanism for thermal dissociation of the Cu, Zn-SOD. However, there were still some shortcomings and difficulties aforementioned in our study on the SOD’ conformation locks.

In the present study, A Steered molecular dynamics (SMD) method was proposed in this paper to study firstly the dissociation of two chain of Cu, Zn-SOD. SMD simulation was been successfully used to investigate ligand–receptor and protein–protein dissociation through the application of external forces^12–23^. By analyzing the energy of SOD during SMD simulation, such as hydrogen bonds, hydrophobic interactions, and others, the key residues to conformation stability of Cu, Zn-SOD were identified. These findings may improve to understand the mechanisms of conformation lock of dimeric proteins (enzymes).

## Results

### Pulling velocity and spring constant

**Figure 1:**
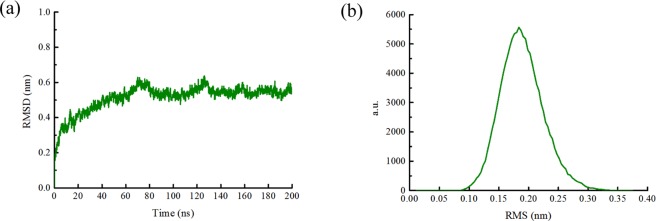
(**a**) The RMSD of the carbon atoms in the protein backbone relative to the initial frame against simulation time. (**b**) RMS distribution of clusters during last 50 ns.

Before SMD simulation, the MD equilibration of the model ran at pH 7 and 300 K. The stability of the model structure could be evaluated by obtaining the value of RMSD and the cluster structures. It could be seen that the structure reached equilibration after about 60 ns and that the relative RMSD value was stable near 0.55 nm (Fig. 1a), and structures had good convergence during last 50 ns (Fig. 1b).

The spring force depended on the spring constant and the tension exerted on the system. The spring force varied greatly in the SMD simulation of fixed speed^24,25^. Higher pulling velocity resulted in loss of equilibration and significant error in simulation.

**Figure 2:**
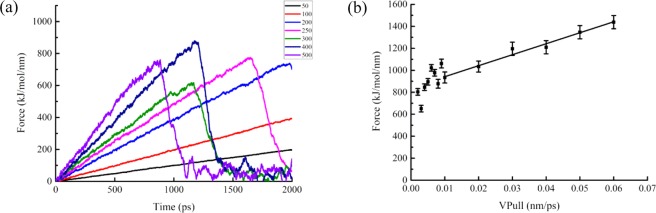
Spring constant and pulling velocity. (**a**) Influence of the spring constant on the steering force of SOD. (**b**) Calculated rupture forces as a function of pulling velocity (Vpull). The error bars give estimated uncertainties.

Figure 2a showed the spring constant effects on steering force of SOD. The rupture force fluctuated greatly when spring constants were larger than 300 kJ mol^−1^ nm^−2^. Meanwhile, effect was not obvious when spring constants were less than 200 kJ mol^−1^ nm^−2^. Therefore, the spring constant of 250 kJ mol^−1^ nm^−2^ was chosen as the best parameter for SOD SMD simulation, and so, the rupture force was measurable and consistent.

The effects of pulling velocity on rupture force were shown in Fig. 2b. It showed that the rupture force had obvious linear correlation when the pulling velocity ranged from 0.001 to 0.08 nm ps^−1^. Therefore, 0.01 nm ps^−1^ (minimum velocity) was used in the subsequent SMD simulation of SOD.

### Energy and conformational changes of SOD during SMD simulation

**Figure 3:**
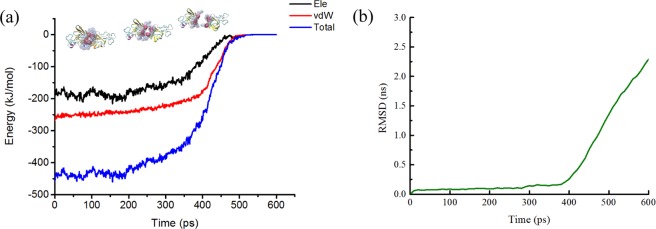
Energy and conformational changes of SOD during SMD simulation. (**a**) Time dependence of the interaction energy between A chain and B chain of SOD. (**b**) The RMSD of the carbon atoms in the protein backbone relative to the initial frame.

The dynamic process of SMD simulation and the change of interaction energy in SOD dissociation process were also demonstrated and recorded under the best simulation conditions mentioned above, and each SMD simulation ran twenty times. The Lennard-Jones–Short Range (LJ–SR) and the Coulombic potential–Short Range (Coul–SR) energies from 0 to 600 ps were being analyzed to get the electrostatic energy and vdW energy. At the starting point (left) of the curve in Fig. 3a, the system was stable with a relatively low free energy. With the beginning of simulation, the system lost its equilibration because of the exerted force. After the starting point, the curve of interaction energy decreased and fluctuated as time went on, which indicated that there were strong interactions between the A chain and the B chain during the dynamic process. The interaction might reduce to zero at the end of the simulation process, indicating completely separation of the B chain of SOD from the A chain. Obvious conformational changes of SOD might take place in the simulation process, and the RMSD value of SMD simulation could also prove these changes (Fig. 3b). Moreover, the distance between the mass center of the A chain and the B chain increased from 2.82 nm at the beginning of simulation to 6.83 nm at the end of simulation.

### Energy changes for hydrogen bonds and hydrophobic bonds during the SMD simulation

**Figure 4:**
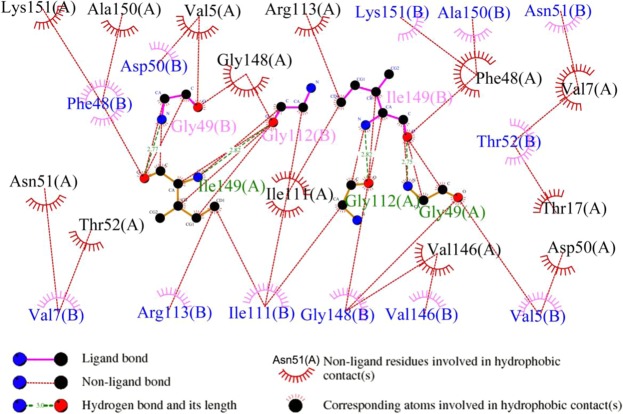
Hydrogen bonds and hydrophobic interactions between A and B chains of SOD. By DIMPLOT programs of LigPlot+ v1.4.5.

**Figure 5:**
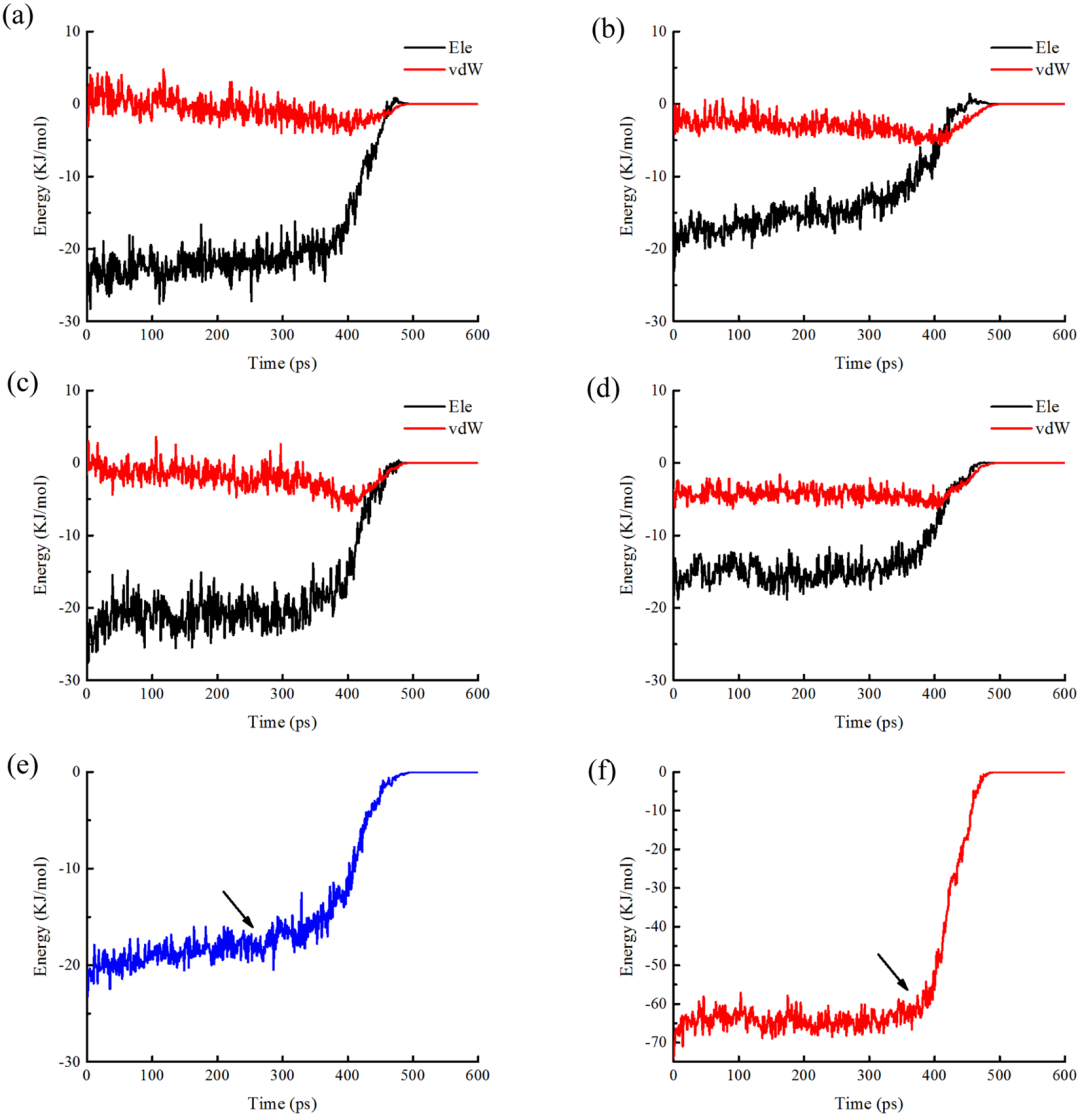
Changes in the interaction energy over time for relevant residues during SMD simulation. (**a**) The interaction energy between Ile149B and Gly49A. (**b**) The interaction energy between Ile149B and Gly112A. (**c**) The interaction energy between Ile149A and Gly49B. (**d**) The interaction energy between Ile149A and Gly112B. (**e**) The total energy between Ile149B and Gly112A. (**f**) The total energy between Ile149B and Gly49A, Ile149A and Gly49B, Ile149A and Gly112B.

Hydrogen bonds and hydrophobic bonds were important stable interactions in protein structure, In order to study the effects of specific residues in the SMD simulation process, the residues of hydrogen bonds and hydrophobic interactions between A and B chains of SOD were generated from LigPlot^+^. Results showed that there were 4 pair hydrogen bonds and 75 hydrophobic bonds (Fig. 4). By analyzing the energy changes during the stretching process, it showed that the energy between residues of hydrogen bonds was mainly composed of electrostatic energy, and there was a small difference in energy between the two pairs of identical residues. Table 1 showed the average interaction energy of hydrogen bonds during the SOD SMD simulation. The energy changes of four pairs of hydrogen bonds in the SMD simulation were shown in Fig. 5. It showed that the energy of hydrogen bond between Ile149B and Gly112A decreased obviously around 270 ps, and then, the energy of the other three hydrogen bonds decreased around 400 ps, afterwards, the energy during the simulation reduced gradually and came to zero at about 520 ps.

**Table 1:** Average interaction energies of Hydrogen-bond residues during SMD simulation.

Residues pair	Electrostatic energy (kJ/mol)	Vdw energy (kJ/mol)	Total energy(kJ/mol)
Ile149A-Gly49B	−14.42	−1.66	−16.08
Ile149A-Gly112B	−10.21	−3.33	−13.54
Ile149B-Gly49A	−15.32	−0.65	−15.97
Ile149B-Gly112A	−9.62	−2.48	−12.10
Total	−49.57	−8.12	−57.69

**Figure 6:**
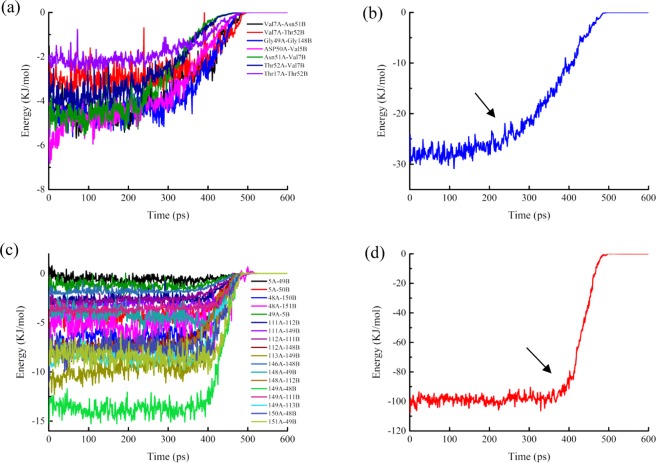
Energy Changes of hydrophobic interactions during SMD simulation. (**a**) The energy changes between 7 pairs of residues. (**b**) The total energy changes between 7 pairs of residues. (**c**) The energy changes between 25 pairs of residues. (**d**) The total energy changes between 25 pairs of residues.

**Figure 7:**
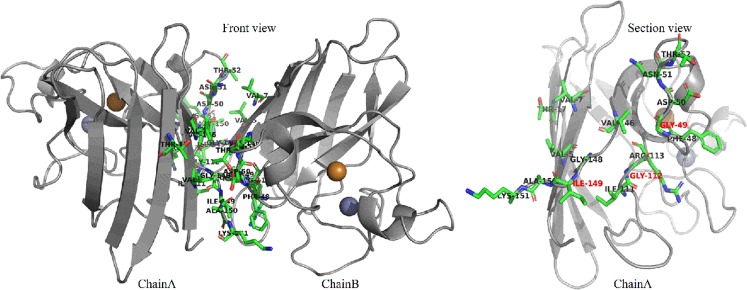
Hydrophobic interactions during the SMD simulation in SOD. Three cores around residues of Gly49, Gly112 and Ile149.

As shown in Table 2, the energy analysis of hydrophobic interactions during the SMD simulations indicated that there were 25 pairs of residues in the interaction between A and B chains of SOD, suggesting that vdW interactions dominated in the most of the residues pairs, and total vdW energy was −74.10 KJ/mol. The total energy of hydrogen bonds was −57.68 KJ/mol. Thus, hydrophobic interactions were important factor in maintaining conformational stability. By analysis of energy of hydrogen bonds and hydrophobic interactions (Fig. 6) during simulation, it was worth noting that the energy of first group of amino acid residues decreased obviously around 240 ps and the second group of 400 ps. Both of them would turn to zero since 520 ps. Combining with the stretch simulation animation, it could be considered that the interaction between the first group of amino acid residues was overcome first, and then the second group (see also Table 3). Combined with the conformational change, these phenomenon indicated that the Gly49, Gly112 and Ile149 played very important role in forming the two groups of the conformational lock of SOD (Fig. 7).

**Table 2:** Average interaction energies of hydrophobic interactions during SMD simulation.

Residues pair	Electrostatic energy(kJ/mol)	vdW energy(kJ/mol)	Total energy (kJ/mol)
Val5A-Gly49B	1.51	−1.94	−0.43
Val5A-Asp50B	−0.25	−2.86	−3.11
Val7A-Asn51B	0.13	−2.96	−2.83
Val7A-Thr52B	−0.02	−2.08	−2.10
Thr17A-Thr52B	0.05	−1.43	−1.37
Phe48A-Ala150B	−1.33	−3.34	−4.67
Phe48A-Lys151B	1.52	−5.42	−3.90
Gly49A-Val5B	1.01	−1.87	−0.86
Gly49A-Gly148B	−0.62	−2.36	−2.97
Asp50A-Val5B	−0.22	−2.65	−2.87
Asn51A-Val7B	0.62	−3.02	−2.40
Thr52A-Val7B	0.03	−2.04	−2.02
Ile111A-Gly112B	−0.07	−1.81	−1.88
Ile111A-Ile149B	−0.42	−1.94	−2.36
Gly112A-Ile111B	−0.05	−1.84	−1.89
Gly112A-Gly148B	−3.45	−1.57	−5.02
Arg113A-Ile149B	−1.01	−5.85	−6.86
Val146A-Gly148B	0.03	−1.31	−1.29
Gly148A-Gly49B	−0.67	−2.49	−3.16
Gly148A-Gly112B	−3.32	−1.81	−5.13
Ile149A-Phe48B	−3.85	−6.17	−10.02
Ile149A-Ile111B	−0.34	−2.32	−2.66
Ile149A-Arg113B	−0.51	−5.39	−5.89
Ala150A-Phe48B	−1.80	−3.83	−5.62
Lys151A-Phe48B	−0.52	−5.83	−6.34
Total	−13.55	−74.10	−87.65

**Table 3:** Two groups of conformation lock.

Group	Amino acid residue pair	Total energy (kJ/mol)
I	Ile149A-Gly112B, Val7A-Asn51B, Val7A-Thr52B, Thr17A-Thr52B, Gly49A-Gly148B, Asp50A-Val5B, Asn51A-Val7B, Thr52A-Val7B	−30.10022723
II	Ile149A-Gly49B, Ile149B-Gly49A, Ile149B-Gly112A, Val5A-Gly49B, Val5A-Asp50B, Phe48A-Ala150B, Phe48A-Lys151B, Gly49A-Val5B, Ile111A-Gly112B, Ile111A-Ile149B, Gly112A-Ile111B, Gly112A-Gly148B, Arg113A-Ile149B, Val146A-Gly148B, Gly148A-Gly49B, Gly148A-Gly112B, Ile149A-Phe48B, Ile149A-Ile111B, Ile149A-Arg113B, Ala150A-Phe48B, Lys151A-Phe48B	−115.234948

**Figure 8:**
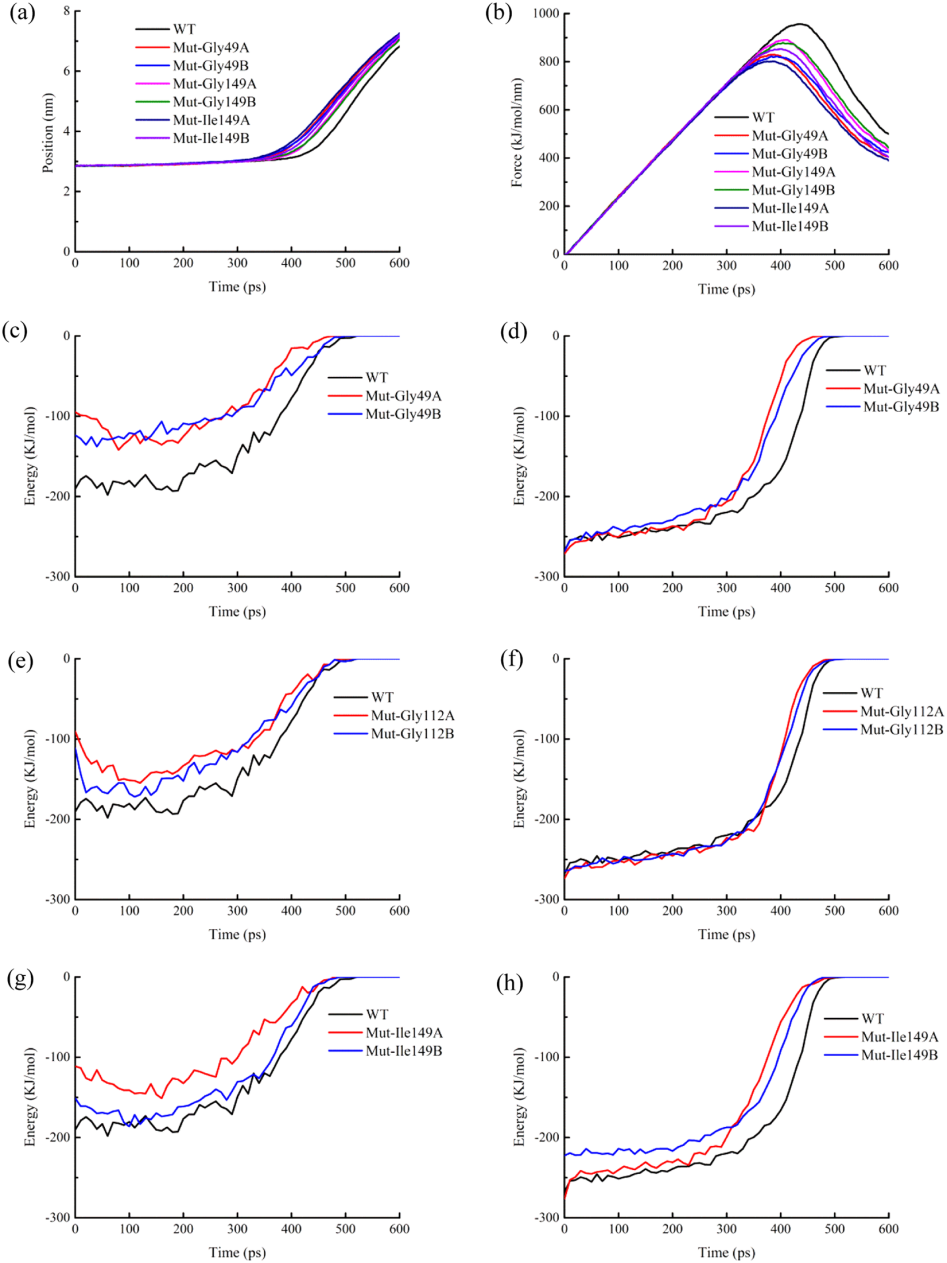
Dissociation of mutants during the SMD simulations. (**a**) COM distance between chain A and chain B of wild type(WT) and six mutants. (**b**) Pull force between chain A and chain B of wild type and six mutants. (**c**) Electrostatic energy of wild type and mut-Gly49. (**d**) vdW energy of wild type and mut-Gly49. (**e**) Electrostatic energy of wild type and mut-Gly112. (**f**) vdW energy of wild type and mut-Gly112. (**g**) Electrostatic energy of wild type and mut-Ile149. (**h**) vdW energy of wild type and mut-Ile149.

To investigate effects the amino acid residues (Gly49, Gly112 and Ile149) on shear force, a series of simple mutants were constructed by using the mutagenesis of Pymol (Mut-Gly49A:Gly49A to Ala49A; Mut-Gly49B: Gly49B to Ala49B; Mut-Gly112A: Gly112A to Ala112A; Mut-Gly112B: Gly112B to Ala112B; Mut-Ile149A: Ile149A to Thr149A; Mut-Ile149B: Ile149B to Thr149B) and executed the energy minimization, equilibration, and the SMD simulations twenty times under the same conditions as mentioned above (see also Fig. 8). The construction of the mutant was based on the following three principles or ideas: 1. Mutants were constructed from hydrophilic amino acid (Gly) to hydrophobic one (Ala), or from hydrophobic amino acid (Ile) to hydrophilic one (Thr). 2. The R group of amino acids had little change. 3. Mutants were obtained by single nucleotide mutation, which might be helpful for future design of molecular biology experiments. Mutants pulled faster under the same conditions. By analyzing the energy of the process between A and B chains, it was shown that the same residues (Gly49, Gly112 and Ile149) on different chains had different function during the SMD simulation. Different behaviors of mutants during stretching revealed that these residues played an important role in maintaining the protein conformation, they were an important part of the conformational lock.

As a special electrostatic interaction, the salt bridge might play an important role in maintaining the conformation stability of a protein. After investigating all the salt bridges in the SOD protein molecule, it could be found that the distance between the amino acid residues (Asp11 Lys9) of the salt bridge between the A and B chains was larger than 0.6 nm from beginning the simulation, so salt bridge had little role in the process of SMD simulation (Fig. S1).

## Discussion

SMD simulations of Cu, Zn-SOD was used to analysis the process of dissociation and conformational lock, by describing the process of energy and conformational changes during the dimeric SOD dissociations. The results suggested that hydrogen and hydrophobic interactions were the main force, and salt bridge had little contribution. By analysis of the trajectory and energy, it was found that the average stretching distance was 0.13 nm near 240 ps, where the energy began to decrease. Within this distance, both hydrogen bonding and hydrophobic interaction maintained the integrity of oligomeric proteins, which were characterized by high stability and thermal denaturation temperature. In general, the energy decreased gradually during the stretching process. Near 400 ps, when the average tensile distance was 0.30 nm, the hydrogen bond began to break, and the energy decreased rapidly. When hydrophobic interaction could not maintain the integrity of the protein, the protein was decomposed into monomers. In combination with previous studies, the essence of SOD conformational lock was hydrogen bonding and hydrophobic interaction. In terms of total energy, the average energy of hydrophobic interaction (−87.65 kJ/mol) was larger than that of hydrogen bond (−57.69 kJ/mol) and played a major role in maintaining conformation of SOD. On the other hand, the average distance between the amino acid centroids of hydrogen bond was 0.19 nm, and the average distance between hydrophobic interactions was 0.36 nm, thus, the role of a single hydrogen bond would be particularly important.

By mutating the amino acid residues that make up the hydrogen bond, the energy of SOD during stretching could be changed and the stability of protein could be affected. In additional, the effects of mutations to Arg, which was highly prevalent in the diseased patients and studies in experimental models were also shown and discussed in Figs S2, S3 and the text in additional document, respectivley. Obviously, the interaction between different amino acid residues, such as Gly49, Gly112 and Ile149, might have important roles in maintaining protein conformation and stability, and these results would be verified by biochemical experiments in mutagenesis in future research. Though energy changes were analyzed through a non-equilibrium way, an umbrella shaped sampling equilibrium dynamic method could also be used for validation study.

Previous literature have shown that the copper and zinc play an important role in maintaining the stability and activity of Cu/Zn SOD. However, this paper mainly studies the interaction of amino acid residues between A and B subunits of SOD to determine which residues in the AB chains play an important role in maintaining the conformation stability of SOD. We call them conformational locks, while copper and zinc are located at the center of the SOD structure, not at the interface of the AB subunit. Therefore, in order to simplify the research process, Cu Zn was removed in the stretching process, but it cannot be denied that Cu Zn plays an important role in maintaining the stability and activity of SOD. At the same time, the phenomenon of subunit deformation is also evident in the stretching process. It may also explain the role of Cu Zn in maintaining protein stability, which can be further discussed in future research.

Generally, SMD simulation might be an efficient way to understand the mechanism of conformation lock of the dimeric proteins.

## Methods

### Model preparation

PDB file of Crystal structure of the Cu, Zn-SOD from bovine erythrocytes (PDB ID:1cbj) was achieved from the Protein Data Bank (http://www.rcsb.org/)^26^. The protein visualization software PyMOL^27^ was applied to remove the ligands (Zn and Cu).

### MD and SMD simulations

The software of Gromacs 2016.4^28–33^ was applied for MD simulations, within the 53a6 force field (FF)^34^. SPC water was adopted to make the protein solvation^35^ in a rectangular cell with dimensions of 84.71 Å × 82.84 Å × 104.95 Å. The chlorine ion (Cl^−^) and sodium ion (Na^+^) were filled to the system to maintain the system’s neutrality by using 100 mM NaCl. The LINCS^36^ was employed to constrain bond length and fix all bonds containing hydrogen atoms. Berendsen thermostat^37^ was chosen to control the temperature at 300 K. The Particle-mesh Ewald algorithm^38,39^ was used to calculate electrostatic interactions with a 10 Å cutoff. The V-rescale and the Parrinello–Rahman algorithms was applied to couple the temperature and pressure. Energy minimization of the system was obtained using steepest descent algorithm with a tolerance value of 1000 kJ mol^−1^ nm^−1^ in 2000 steps. After energy minimization, NVT and NPT equilibrations were acted on the system for 2 ns. Production MD was performed for 200 ns time duration for the simulations at 300 K temperature and 1 bar pressure.

The initial configuration of the pulling simulation started with the structures of the MD simulation trajectory. The structure was settled down a rectangular box of 84.71 Å × 82.84 Å × 170.15 Å. As that mentioned above, the simulation cell was filled with SPC water and 100 mM NaCl. The pulling simulations were implemented by running the GROMACS 2016.4 to take advantage of pull code improvements and new features implemented during the course of the project. By using aforementioned method, equilibration took 300 ps under a NPT ensemble. A dynamic snapshot of the conformation and steering force was recorded every 0.1 ps and 10 fs, respectively. The stretching time was 2 ns. One of the animations was shown in the additional video. The B chain of SOD was pulled away from the A chain with a pulling force at a constant speed (v) according to Eq. 5^16,40^:

5 $${\rm{F}}=-\,{\rm{k}}\,[{{\rm{x}}}_{{\rm{pull}}}({\rm{t}})-{{\rm{x}}}_{{\rm{pull}}}(0)-{\rm{vt}}]$$

where F, k, v, x_pull_(t) and x_pull_(0) were the pulling force, spring constant, the positions of the pulled atom at the time t and at the initial time, respectively.

### Trajectory analysis

Applications in the Gromacs package were applied to analyze the obtained trajectories. The root mean square deviation (RMSD) was calculated using gmx rms. The energy extracts were using gmx energy and compute salt bridge was performed using the GROMACS tool gmx saltbr. Twenty times simulations were repeated at a random seed number and the results were analyzed by excel. VMD^41^ and LigPlot^+^^42^ were applied to the visualization and analysis of the stretching process, respectively.

## Supplementary information

stretching animation

additional document
