# Supplementary material for: Steered molecular dynamic simulations of conformational lock of Cu, Zn-superoxide dismutase
Source: Sci Rep. 2019 Mar 13;9:4353. doi: 10.1038/s41598-019-40892-0 (PMC6416402; doi:10.1038/s41598-019-40892-0)
Supplement: Supplementary file 2 — additional document [file 41598_2019_40892_MOESM2_ESM.docx]

**Steered molecular dynamic simulations of conformational lock of Cu,Zn-superoxide dismutase**

Bao-Lin Xiao^1#^, Yan-Na Ning^1#^, Nan-Nan Niu^1#^, Di Li^1#^, Ali Akbar Moosavi-Movahedi^2^, Nader Sheibani^3^, Jun Hong^1#,4,*^

^1^School of Life Sciences, Henan University, JinMing Road, Kaifeng 475000, China

^2^Institute of Biochemistry and Biophysics, University of Tehran, Enquelab Avenue, P.O. Box 13145-1384, Tehran, Iran

^3^ Department of Ophthalmology and Visual Sciences and Biomedical Engineering, University of Wisconsin, School of Medicine and Public Health, Madison, WI 53726, USA

^4^Henan Engineering Laboratory for Mammary Bioreactor, School of Life Sciences, Henan University JinMing Road, Kaifeng 475000, China

* Corresponding author

E-mail: hongjun@henu.edu.cn (J. Hong)

^#^ These authors contributed equally to this work.

**Additional document**





**Fig S1 Role of salt bridge in the process of SMD simulation**

**
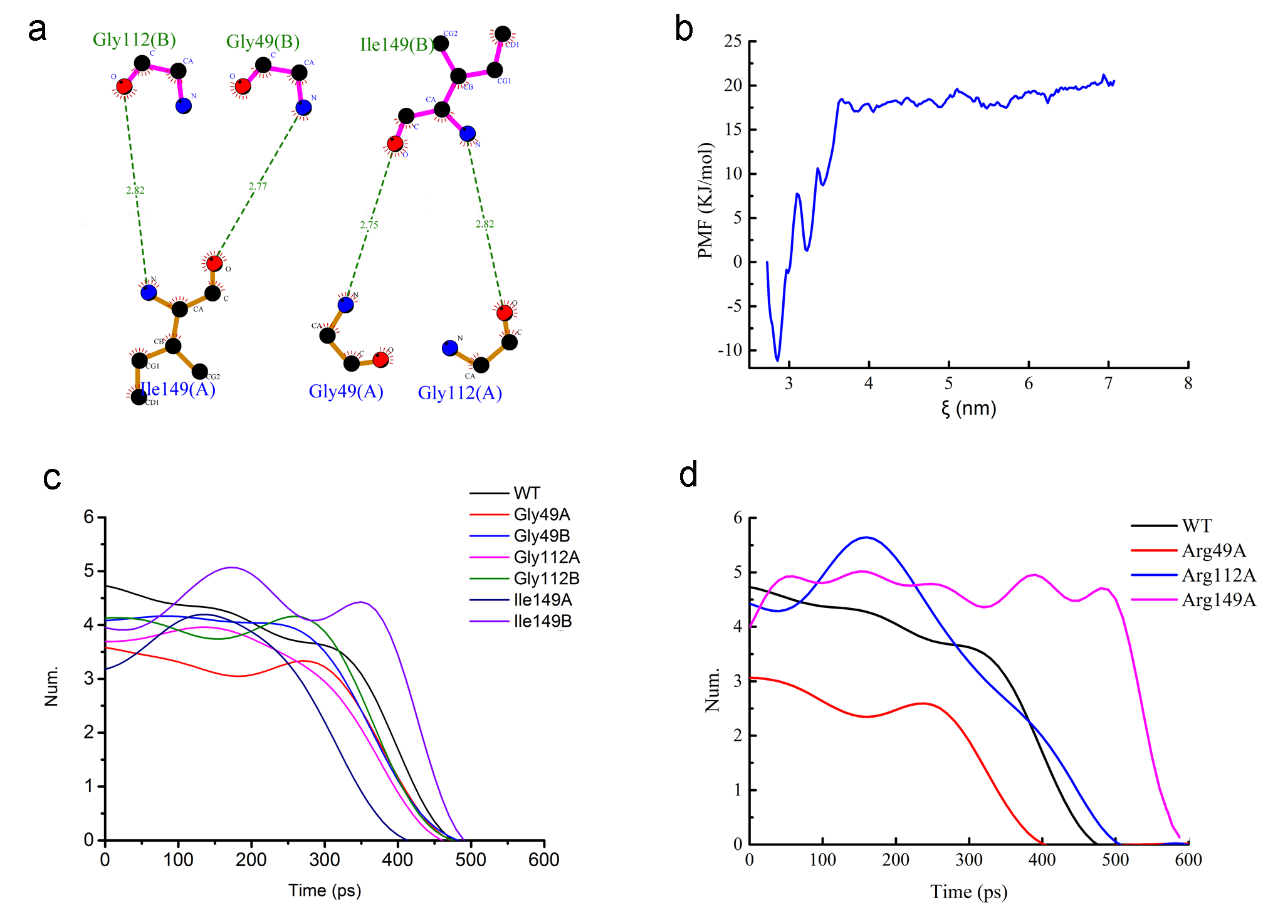
**

**Fig S2 The changes of hydrogen bonds during the SMD simulation**

a. Four pair residues of hydrogen bonds

b. Potential of Mean Force of SOD

c. Number of hydrogen bonds of WT and six mutants

d. Number of hydrogen bonds of WT and Arg mutants





**Fig S3 Changes in the interaction energy over time for relevant residues during SMD simulation.**

a.c. e. The electrostatic energy changes of WT and mutants

b .d. f. The vdW energy changes of WT and mutants

The amino acid residues that form hydrogen bonds between subunits A and B have both hydrogen bonding and non-bonding interactions. The energy analysis shows that the electrostatic force conforms to the law of bond length. During the stretching process, the number of hydrogen bonds decreases as the subunits are pulled apart. Figure S2b shows that ΔG = 29.43 kcal/mol was calculated by Weighted Histogram Analysis Method (WHAM), and considering particularly that there are two defects (3.23,3.38 nm) in the PMF profile and this regions are a high-energy state.

Through Figure S2, Figure S3, Figure 8, it can be found that a hydrogen bond (bond length 2.75) breaks in the first region, and the non-bond interaction also decreases in tension at the same time. Then the second hydrogen bond (bond length 2.77) broke, and soon the other two hydrogen bonds (bond length 2.82) also broke, and the two subunits of AB were pulled apart. Through figure S2, figure S3, figure 8, it can be found that mutation of key amino acids will affect both hydrogen bond and non-bond interactions, thus reducing the interaction energy between AB subunits and reducing the stability of proteins. It should be pointed out that the interaction between two subunits may be enhanced by the Arg mutation Ile149A, due to the large variation of R base, which may enhance the stability of protein. It still needs further experiments to verify.
